# Cervical dysplasia after renal transplantation: A retrospective cohort study

**DOI:** 10.4274/tjod.galenos.2021.28938

**Published:** 2021-03-12

**Authors:** Ahmet Bilgi, Şevki Göksun Gökulu, Orkun İlgen, Mehmet Kulhan, Seda Akgün Kavurmacı, Hüseyin Toz, Mustafa Coşan Terek

**Affiliations:** 1 Selçuk University Faculty of Medicine, Department of Gynaecology and Obstetrics, Konya, Turkey; 2 Mersin University Faculty of Medicine, Department of Gynaecology and Obstetrics, Mersin, Turkey; 3 Dokuz Eylül University Faculty of Medicine, Department of Gynaecology and Obstetrics, İzmir, Turkey; 4 Ege University Faculty of Medicine, Department of Gynaecology and Obstetrics, İzmir, Turkey; 5 Ege University Faculty of Medicine, Department of Internal Medicine, İzmir, Turkey

**Keywords:** Immunosuppression, cervical dysplasia, renal transplantation

## Abstract

**Objective:**

Since the first days of organ transplantation, it has been accepted that solid transplant recipients have a high risk of developing cancer. Chronic immunosuppression and environmental factors play a role in cancer development in recipients. In the present study, we tried to evaluate the cumulative incidence of cervical dysplasia after renal transplantation, risk factors for disease development, and the time until high-grade dysplasia occurred.

**Materials and Methods:**

A total of 50 patients with renal transplantation who presented for gynecologic follow-up was included in the study. The medical records of the patients were reviewed until the last clinical visit, their demographic characteristics, transplant history, gynecologic history, and gynecologic examination results (cervical cytology and histology reports) were reviewed.

**Results:**

Of the 50 women in the study population, 29 (58%; 95% confidence interval: 8.8-15.9) developed cervical dysplasia after the first transplant at a median follow-up of 7.8 (range: 4.6-12.9) years. Twenty-one women with benign cervical cytology before transplantation had evidence of low-grade intraepithelial lesions + after transplant (47% of these were within 2 years after transplant). During the follow-up, 8 women (18.2%) were diagnosed as having high-grade intraepithelial lesions + (within 5 years after transplantation).

**Conclusion:**

Renal transplant patients were found to have higher abnormal cervical cytology and histology rates than the normal population.

**PRECIS:** Incidence of cervical dysplasia increased in patients with renal transplant.

## Introduction

Primary (*de novo*) malignancy that develops after renal transplant is seen as an unfortunate complication of a successful surgery. The development of such malignancies may be caused by various factors such as individual and regional susceptibility, pre-transplant disease states, viral status of the recipient, and high doses of various immunosuppressive drugs to protect the graft. Lymphoproliferative disease, skin cancer, Kaposi’s sarcoma, and cervical dysplasia have been reported with high incidence in these patient groups after transplantation^(1)^. The persistence of human papillomavirus (HPV) is the most important factor in the development of dysplasia. Therefore, if immunologic control of the virus is interrupted, this adverse event poses a higher risk to patients. Immunocompromised women are at high risk for a variety of premalignant and malignant conditions, including cervical dysplasia^(2)^. There are many factors in the development of cervical malignant lesions but immunosuppression on high-risk HPV clearance is the most important^(2,3)^. The long-term consequences of the suppressed immune system are becoming increasingly important for improving life expectancy and quality. Cervical cancer screening guidelines are constantly updated due to the increase in knowledge about the etiopathogenesis of the disease; however, the ideal method and frequency for cervical cancer screening after organ transplantation is still uncertain. Therefore, in this study, we aimed to evaluate the abnormal findings resulting from cervical cancer screening and histology in women with renal transplantation. We tried to evaluate the cumulative incidence of cervical dysplasia after renal transplantation, to determine risk factors for disease development, and to evaluate the time to high-grade dysplasia. In addition, we aimed to determine whether the risk of abnormal cervical testing approached the general population risk. The point we wanted to draw attention to in our study was to emphasize the factors that might be cofactors of cervical dysplasia and to stress the importance of multidisciplinary evaluation of patients with organ transplants who are difficult to follow-up.

## Materials and Methods

We retrospectively reviewed the patient records. The study was approved by the ethics committee of our university (no: 99166796-050.06.04, approval no: 20-8.1T/8). Patients with renal transplant who presented for gynecologic follow-up were determined from our hospital’s database. Written informed consent was obtained from participants to use their medical records for research. As the inclusion criteria, it was decided to have documented gynecologic follow-up for at least 1 year after renal transplant and to have at least one cervical pathology sample before or after transplant. Patients who had a hysterectomy prior to transplantation or within 1 year were excluded. After applying the inclusion and exclusion criteria, a total of 50 patients were included in the study. The medical records of the patients were reviewed until the last clinical visit, and demographic characteristics, transplant history, gynecologic history and results of gynecologic exams (cervical cytology and histology reports) were reviewed.

We created three categories according to the cytologic and histologic features:

1. Cervicitis, inflammation, atypical squamous cells of undetermined significance (similar lesions were classified as benign)2. Low-grade intraepithelial lesions (LSIL), cervical intraepithelial neoplasia (CIN) I were classified as LSIL+3. High-grade intraepithelial lesions (HSIL), CIN II, and CIN III were classified as HSIL+

Descriptive statistics were reported as means and standard deviations (SD), and interquartile medians, frequencies, and percentages as indicated. The cumulative incidence of cervical dysplasia in the general population was calculated based on the gold standard tissue diagnosis. Findings of abnormal screening tests (based on clinical documentation or pathologic diagnosis) have also been reported. Patient demographics such as age, transplant age, body mass index (BMI) (weight in kilograms divided by height in square meters), parity, immunosuppression agent, and dialysis type and time status are tabulated.

### Statistical Analysis

Data are presented as the number of observations (n, %), mean ± SD, range. The results of homogeneity (Levene’s test) and normality (Shapiro-Wilk test) were used to decide the statistical methods for comparing the study groups. Among normally distributed groups with homogeneous variances, dependent groups were compared using Student’s t-test. According to the test results, parametric test assumptions were not available for some variables; therefore, the independent groups were compared using the Mann-Whitney U test. Categorical data were analyzed using Fisher’s Exact test and the chi-square test. In cases in which the expected counts for inclusion were not met in less than 20% of the cells, Monte Carlo simulation was used and the values ​​were determined. Logistic regression analysis was performed to determine whether cervical dysplasia was positive or negative. Statistical analyses were performed using the IBM SPSS Statistics for Windows Version 25.0 software package (Armonk, NY: IBM Corp). P-values <0.05 were considered statistically significant. Nonparametric cumulative incidence analyses were performed using the Stata 11.0/MP for Linux package (StataCorp, College Station, Tex). Non-parametric cumulative incidence estimates were produced using the stcompet command, and multivariate comparisons of cumulative incidence functions were completed using stcrreg.

## Results

A total of 50 women with renal transplantation were evaluated for gynecologic follow-up and cervical screening. Transplantation was performed when the patients were aged 41.6-14.2 years on average, and gynecologic follow-up was started at least 1 year before transplantation. The mean follow-up period was 5.07 (4.98) years in patients with cervical dysplasia and 6.76 (4.72) years in patients without dysplasia. The most common renal diseases were diabetes mellitus (66%), hypertension (14%), and lupus nephritis (10%). For 11 (22%) patients, the diagnosis was other renal diseases. Table 1 summarizes the demographic and basic characteristics of the patients. The median age of these 50 women was 44.55 in the group that developed cervical dysplasia and 44.67 in the group that did not develop cervical dysplasia [interquartile range (IQR): 29-53, 24-36, respectively] (Table 1). Of the 50 women in the study population, 29 [58%; 95% confidence interval (CI): 8.8-15.9] developed cervical dysplasia after the first transplant at a median follow-up of 7.8 years (IQR: 4.6-12.9). Forty-four women (88%) had at least one documented benign cervical pathology prior to renal transplant. All patients had at least one documented cervical screening report after renal transplant.

**Figure 1:**
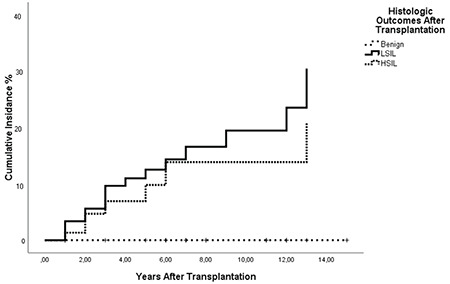
Cumulative incidence rates for LSIL+ and HSIL+ cytology-histology for the cohort LSIL: Low-grade intraepithelial lesion, HSIL: High-grade intraepithelial lesion

Twenty-one women with benign cervical cytology before transplantation had evidence of LSIL+ after transplant (47% of these were within 2 years after transplant). During the follow-up, eight women (18.2%) were diagnosed as having HSIL+ (within 5 years after transplantation). Table 2 and Figure 1 show the cumulative incidence rates for LSIL+ cytology-histology,  HSIL+ cytology-histology for the cohort.

Factors associated with an increased risk of developing cervical dysplasia in univariate and multivariate analysis were: age [Odds ratio (OR)=1.22, 95% CI: 0.395-3.770], time after transplant (OR=1.007, 95% CI: 0.929-1.091), the use of cyclosporine-A (OR=1.381 95% CI: 0.554-2.336), the use of tacrolimus (OR=1.731 95% CI: 0.224-2.382), the use of azathioprine (OR=1.893, 95% CI: 0.268-2.971), the use of mycophenolate mofetil (OR=2.184, 95% CI: 0.101-47.266), hemodialysis (OR=1.25, 95% CI: 0.292-5.348), peritoneal dialysis (OR=1.5, 95% CI: 0.255-8.817), and hemodialysis + peritoneal dialysis (OR=1.851, 95% CI: 0.218-9.695). Among the factors that reduce the risk of the development of cervical dysplasia were the following parameters: age at transplantation (OR=0.817, 95% CI: 0.261-2.557), years of follow-up (OR=0.689, 95% CI: 0.340-1.398), current smoker or quit within the past 1 year (OR=0.214, 95% CI: 0.045-1.032), and BMI (OR=0.847, 95% CI: 0.712-1.006). Table 3 shows the univariate and multivariate analyses of factors associated with cervical dysplasia.

## Discussion

After the spread of organ transplantation worldwide, lymphoid and non-lymphoid tissue malignancies, especially skin cancers, have started to be seen in organ transplant recipients with a high incidence. Organ transplant recipients are at a 3- to 4-fold risk of malignancy due to chronic immunosuppression. However, compared with the general population, the relative risk for certain cancers increases 100-fold^(4)^. The risk of malignancy is estimated as 20% after 10 years of chronic immunosuppression^(5)^. Possible mechanisms for malignancy development include replication of oncogenic viruses (HPV, herpes simplex virus, Epstein-Barr virus, cytomegalovirus), immunity disorders (suppression of natural killer cell activity, impairment of immune regulation, use of blood products, decrease in interferon levels), and direct carcinogenic effects of immunosuppressives^(6)^. The most important risk for cervical cancer is infection with high-risk HPV types^(7)^. The most common sexually transmitted disease seen in the general population and patients with renal transplantation is HPV infection. Patients with renal transplants have a higher rate of permanent disease and disease burden compared with the general population^(8)^. It was reported that the risk of developing cervical neoplasia (usually *in situ*) was 14 times higher in female patients who had renal and liver transplantation compared with controls^(9)^. Chapman and Webster^(10)^ reported that 46 of 13,077 patients (6.6%) who had a renal transplant were diagnosed as having cervical cancer. It has been reported that the incidence of primary (*de novo*) malignancy after organ transplantation is between 2.92% and 3.36% in renal transplant recipients in Turkey^(11)^. Haberal et al.^(12)^ reported that the incidence of malignancy among renal transplant recipients was 3.7%, a 47-year-old female patient was diagnosed as having cervical cancer 4 months after renal transplant, and the incidence of gynecologic malignancy was 2% among renal transplant recipients^(12)^. Akgun et al.^(13)^ reported that of 347 renal and 24 liver transplants performed in Organ Transplantation Centers, malignancy developed in 15 renal transplant patients (3.36%) and one liver transplant patient (3.84%) during 13 years of follow-up, and one in situ cervical carcinoma developed 4 months after renal transplantation^(13)^. It has been reported that the HPV prevalence can be as low as 5% and as high as 63% in studies of female kidney transplant patients^(14)^. The risk of persistent infection with HPV type 16-18 genotypes is higher in immunocompromised patients than in the general population^(8)^. Similarly, in another study conducted in patients with kidney transplantation, although the incidence of HPV-related malignancy was found to be increased after transplantation, the same increase was not observed in patients who developed end-stage renal failure but who are not currently transplanted^(15)^. These findings support the role of immunosuppressive agents in increasing the risk of HPV-related diseases in patients with renal transplantation.

In our clinic, a total of 50 female patients underwent renal transplantation between 2016-2017 and received immunosuppressive therapy. Of the 50 women in the study population, 29 (58%; 95%: CI 8.8-15.9) developed cervical dysplasia after the first transplant at a median follow-up of 7.8 years. Twenty-one women with benign cervical cytology before transplantation had evidence of LSIL + after transplant (47% of these were within 2 years of transplantation). During the follow-up, eight women (18.2%) were diagnosed as HSIL + (within 5 years after transplantation). Cervical cancer was not detected in any patients in our study.

There are also studies reporting that there is no increase in the risk of developing gynecologic malignancy after organ transplantation, on the contrary, the relative frequency of gynecologic tumors decreased compared with the general population. Fung et al.^(16)^ reported that gynecologic malignancies (breast, ovary, uterus and cervix) in women who underwent organ transplantation were 1.9 times less frequent than in the normal population and concluded that this was due to the active mammographic and gynecologic examination policy before and after liver transplantation. In a study conducted among 1,778 patients who underwent organ transplantation in the United Kingdom, it was reported that cervical cancer was detected in one of 78 women who developed primary (*de novo*) non-lymphoid tissue malignancy, the expected incidence of cancer in terms of cervical cancer was 0.79%, and no increased risk of cervical and breast cancer was observed^(17)^. However, the role and extent of immune dysfunction in the development of cervical dysplasia are not clear in this patient population. In a study evaluating the incidence of cervical cancer after transplantation, a similar incidence was found in groups with and without systemic lupus erythematosus (SLE); however, specific immunosuppressive drugs or the severity of SLE have not been evaluated^(18)^.

In our study, 0.71% of patients developed LSIL+, and 0.33% developed HSIL or worse lesions within the first 5 years after transplantation. In a study examining the relationship between organ transplantation and invasive cervical cancer, an average interval of 3.8 years was found between transplant and cancer^(19)^. In our study, we saw that a significant number of LSIL+ or worse lesions occurred within 5 years after transplantation; this increases the importance of annual screening especially in this patient group in the first 5 years. The 5-year cumulative HSIL+ incidence in our study was 0.33%, similar to the 0.3% reference cohort rate^(20)^. Our cohort consisted of women with lower levels of abnormal cytology, LSIL+ and HSIL+ histology, compared with a cohort of about one million women^(21)^.

The treatment of primary (*de novo*) malignancies in renal transplant patients is the same as in normal non-transplant patients. In patients with immunosuppressive therapy, especially with solid organ transplantation, Papanicolaou (PAP) tests can be obtained at the first examination, if there is a positive PAP test, examination with colposcopy and biopsy should be taken from these lesions in the presence of suspicious lesions^(22,23)^. However, in these patients, the dose of immunosuppression is reduced to the lowest possible level immediately after tumor diagnosis. It is very important to diagnose and stage malignancies in organ transplant recipients as soon as possible. When these lesions are detected as either *in situ* or low-grade malignancies, oncologic results are undoubtedly better.

### Study Limitations

Our study has some limitations. First, it has a retrospective design and the second is that it is conducted in a single institution. Our HPV data, which are the most important limitation of our study, are limited in this analysis, so we could conclude about how the HPV test might affect the screening range in this population.

## Conclusion

Renal transplant patients have been found to have higher abnormal cervical cytology and histology rates than the normal population. Female patients undergoing organ transplantation should be screened for cervical cancer with annual PAP-smear tests and pelvic examinations. Organ recipients at high risk for malignancy (those with a history of cancer or an underlying disease predisposing to malignancy) should be followed up closely, including colposcopy.

## Figures and Tables

**Table 1:**
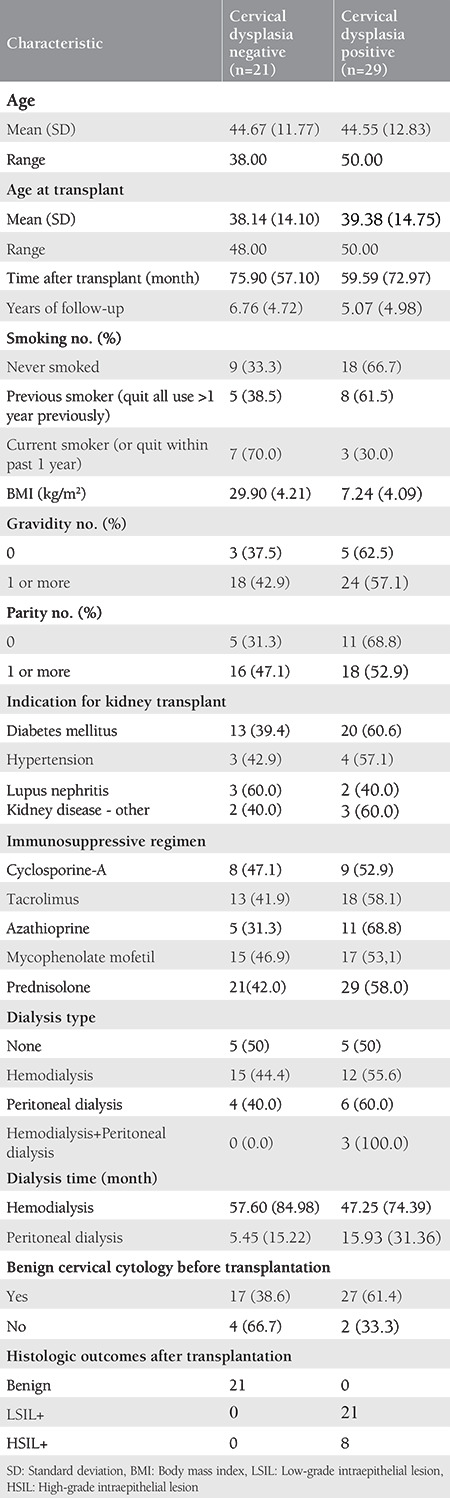
Demographic characteristics of patients

**Table 2:**
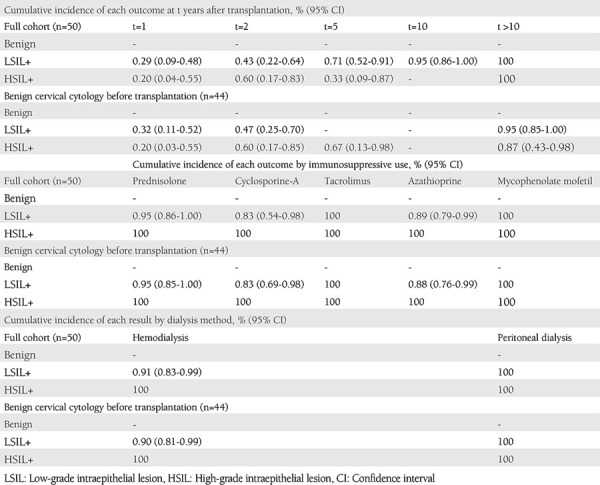
Histologic outcomes after transplantation

**Table 3:**
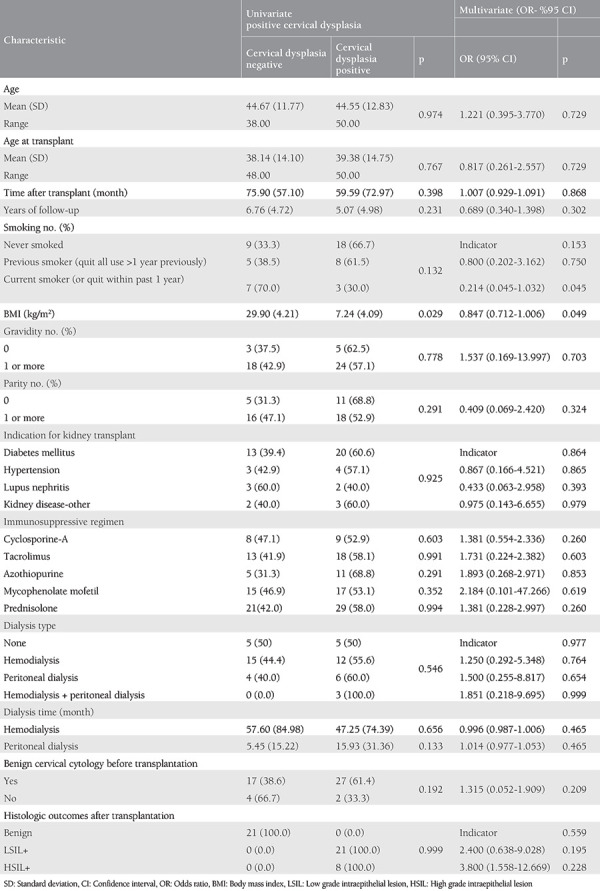
Univariate and multivariable analysis: associations with positive cervical dysplasia
